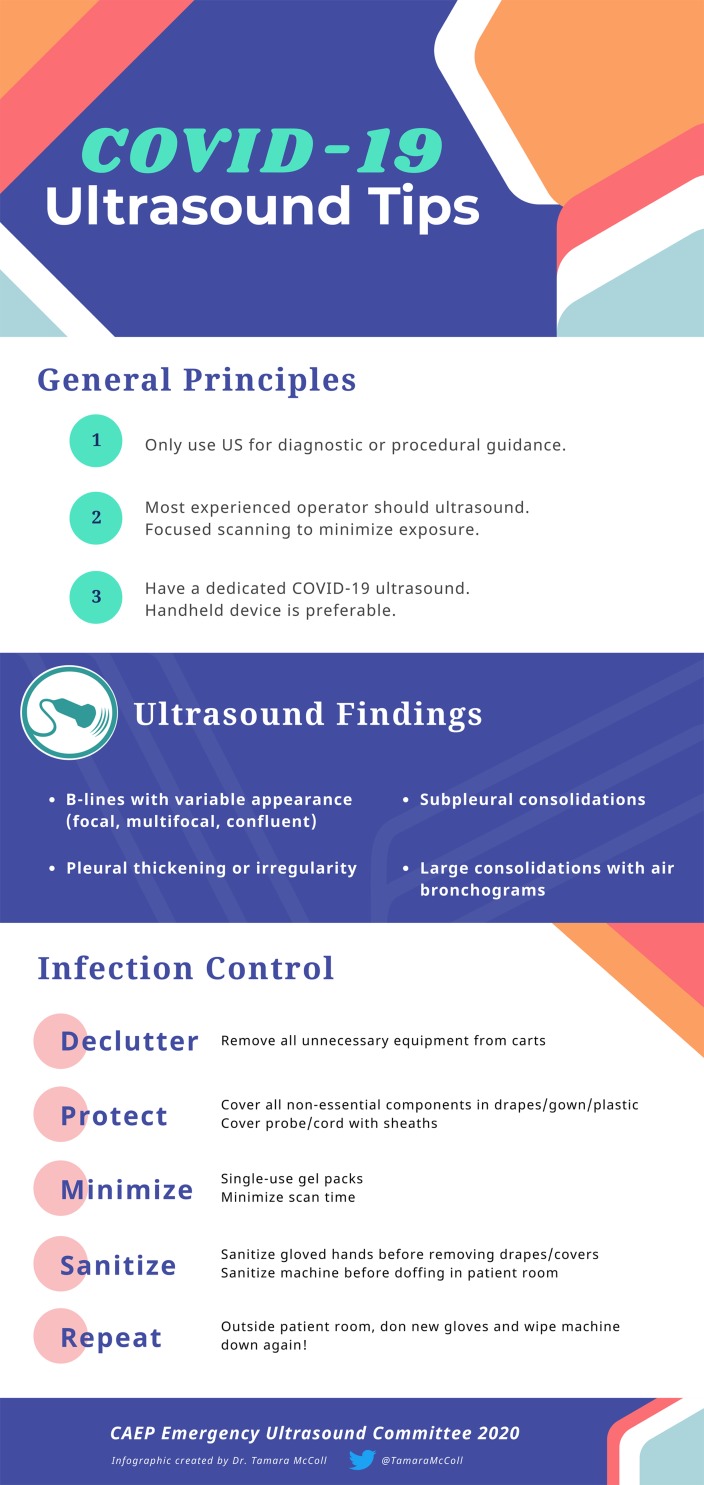# Just the Facts: Recommendations on point-of-care ultrasound use and machine infection control during the coronavirus disease 2019 pandemic

**DOI:** 10.1017/cem.2020.364

**Published:** 2020-04-09

**Authors:** Daniel J. Kim, Tomislav Jelic, Michael Y. Woo, Claire Heslop, Paul Olszynski

**Affiliations:** * Department of Emergency Medicine, University of British Columbia, Vancouver, BC; † Department of Emergency Medicine, Vancouver General Hospital, Vancouver, BC; ‡ Department of Emergency Medicine, University of Manitoba, Winnipeg, MB; § Department of Emergency Medicine, University of Ottawa and Ottawa Hospital Research Institute, Ottawa, ON; ¶ Division of Emergency Medicine, Department of Medicine, University of Toronto, Toronto, ON; ** Department of Emergency Medicine, University of Saskatchewan, Saskatoon, SK

**Keywords:** COVID-19, emergency medicine, pandemic, POCUS

## Abstract

The World Health Organization declared the novel coronavirus disease 2019 (COVID-19) to be a pandemic on March 11, 2020, and, currently, there are over 10,000 confirmed cases in Canada, with this number expected to grow exponentially. There has been widespread interest in the use of point-of-care ultrasound (POCUS) in the management of patients with suspected COVID-19. The CAEP Emergency Ultrasound Committee has developed recommendations on the use of POCUS in these patients, with an emphasis on machine infection control measures.

## INTRODUCTION

The World Health Organization declared the novel coronavirus disease 2019 (COVID-19) to be a pandemic on March 11, 2020, and, currently, there are over 10,000 confirmed cases in Canada, with this number expected to grow exponentially. There has been widespread interest in the use of point-of-care ultrasound (POCUS) in the management of patients with suspected COVID-19. The CAEP Emergency Ultrasound Committee has developed recommendations on the use of POCUS in these patients, with an emphasis on machine infection control measures.

## CASE

A 67-year-old male presents to the emergency department (ED) with a 5-day history of fevers and chills, nonproductive cough, myalgias, and malaise. He has a past medical history of hypertension and dyslipidemia. Of note, he was at a dental conference 2 weeks prior where an attendee had tested positive for COVID-19. At triage, his temperature is 38.7°C, heart rate 120, blood pressure 80/40, respiratory rate 24, and oxygen saturation 93% on room air. The triage nurse fits the patient with a mask, places him in an individual room, and puts him on contact and droplet precautions.

## KEY CLINICAL QUESTIONS

*1.*: ***What are the clinical indications for the use of POCUS in a patient with known or suspected COVID-19?***

*Answer:* Radiographic findings in COVID-19 are nonspecific, so it is unclear whether POCUS has a role in the screening of clinically well patients with suspected COVID-19. These patients should be tested according to your local public health guidelines or instructed to self-isolate for 2 weeks if not tested.

POCUS should only be used if it is expected to change clinical management, like a case of a critically ill patient with undifferentiated hypotension or dyspnea or to aid procedural guidance. It should be used by the most experienced operator involved in the care of the patient, and scanning time should be minimized as much as possible.

At the current moment, there has been no clear determination of which scanning technique is best, or how clinically important the lung ultrasound findings are. It is also not known whether there is any relationship between the severity of POCUS findings and a patient's clinical course.

*2.*: ***What are typical lung and cardiac ultrasound findings in COVID-19?***

**Figure 1.**
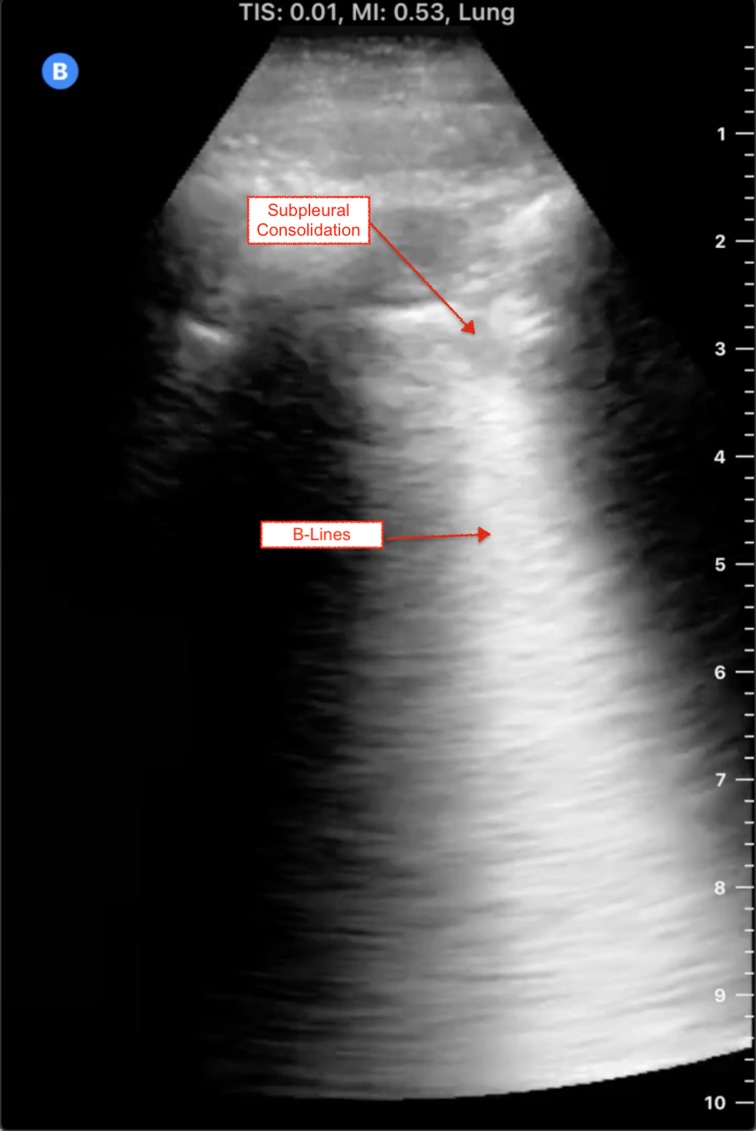
COVID-19-positive patient's lung ultrasound findings.

*Answer:* Current evidence is limited to a small number of studies with small numbers of patients. Prior to scanning, ensure that your machine is set to a lung preset. If your ultrasound machine does not have a lung preset, use the abdominal or cardiac preset with tissue harmonic imaging and multi-beam (MB) settings turned off. By turning off these settings, ultrasound artefacts, which are the basis of lung ultrasound, will become more prominent. Patients with COVID-19 commonly have lung ultrasound findings: B lines with variable appearance (including focal, multifocal, and confluent), subpleural consolidations (Figure 1), pleural thickening or irregularity, and larger consolidations with occasional air bronchograms. A multi-lobar distribution of abnormalities is common. Ultrasound B lines correspond to the typical ground glass opacities visualized on CT imaging. Pleural effusions, other than small localized effusions, are rare. Abnormalities are most commonly visualized in the posterior and inferior lung fields.^1–3^

In the early stages of infection, the main ultrasound finding is focal B lines. As the disease progresses, B lines can become multifocal and confluent, with further development into frank consolidations.^1–3^ Early lung ultrasound findings of B lines and subpleural consolidation can be seen here: https://youtu.be/fO3fFsZQjUg.

COVID-19 patients may also develop severe cardiac complications, including heart failure, myocarditis, and cardiogenic shock. Point-of-care echocardiography may demonstrate ventricular dilatation or reduced left ventricular ejection fraction.^4^

*3.*: ***What is the most appropriate infection control strategy for using ultrasound to minimize the risk of transmission of COVID-19?***

*Answer:* If possible, an ED should designate a specific ultrasound machine for suspected COVID-19 patients. Handheld ultrasound devices should preferentially be used, as they can be completely encased with a probe cover, can be easily cleaned, and do not have a cooling fan.^3,5^ If a handheld device is not available, cart-based ultrasound machines should be stripped of all unnecessary items, like printers, baskets, and gel bottles. Machines with touch screens are preferable to machines with keyboards or buttons. Employ single-use gel packets instead of gel bottles.

The U.S. Environmental Protection Agency (EPA) maintains an updated list of disinfectants for use against COVID-19: https://www.epa.gov/pesticide-registration/list-n-disinfectants-use-against-sars-cov-2. Health Canada also maintains a similar list: https://www.canada.ca/en/health-canada/services/drugs-health-products/disinfectants/covid-19/list.html. Most ultrasound manufacturers are waiving rules about machine-specific disinfectants and will support the use of any product effective against COVID-19. All cleaned surfaces should be fully wet for the product's required contact time.

*4.*: ***What are the appropriate steps for safely donning and doffing the ultrasound machine when scanning a patient with known or suspected COVID-19?***

*Answer:*

**Figure 2.**
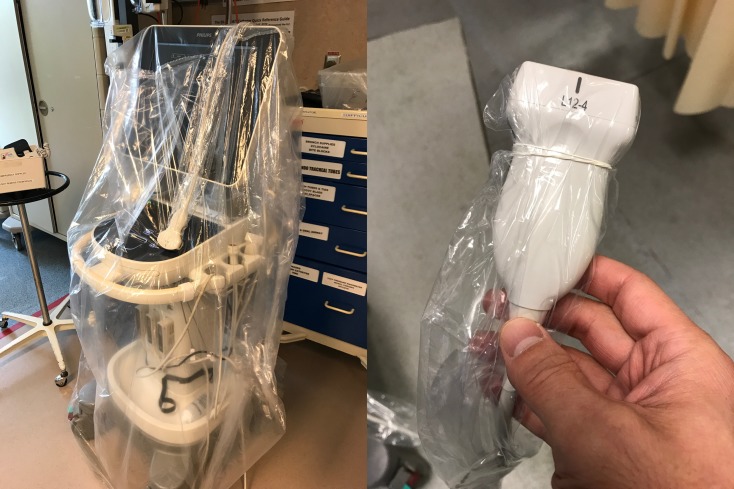
With AGMP, cover non-essential components of the ultrasound machine with drapes, gown, or plastic covering, and use sheaths to cover the probe and cord.

1. For the duration of the pandemic, remove all unnecessary equipment from your cart-based machines.*
2. Consider designating certain machines as COVID-19-specific.
3. If in a higher risk setting with aerosol generating medical procedures (AGMP), cover non-essential components of the ultrasound machine with drapes, gown, or plastic covering, and use sheaths to cover the probe and cord. These do not need to be sterile in the setting of diagnostic applications (Figure 2).
4. Use single-use ultrasound gel packets.
5. Perform the scan.
6. After completion of the scan, sanitize gloved hands while in the patient's room.
7. Remove drapes and coverings after use and leave in the patient's room, being mindful to not disperse viral contaminants while disposing of the cover.
8. Wipe down the entire surface of the machine with disinfectant wipes while inside the patient's room, prior to doffing. Allow surfaces to dry.
9. Move the machine out of the patient's room. Doff contaminated personal protective equipment (PPE) and don new gloves. Then again wipe down the entire surface of the machine.

*For handheld devices, apply the same steps, keeping in mind that a single sleeve should properly cover the entire device. In higher risk settings (like AGMP), hand the device to a runner for a second cleaning.

*5.*: ***How can POCUS programs continue to deliver and maintain ultrasound training during the COVID-19 pandemic?***

*Answer:* POCUS, by its nature, is hands on, requiring a large number of practice scans to achieve competence. Such contact with patients is usually safely managed with standard equipment cleaning protocols but is virtually impossible during pandemic times given strains on PPE, cleaning supplies, and clinician time. Programs should suspend such POCUS training for the duration of the pandemic.

However, programs can still deliver POCUS education in the form of image review, simulation, phantoms, case-based modules, and online learning. While this type of learning cannot replace practice scans on actual patients, it reinforces the key skills of image interpretation and clinical integration into medical decision-making. Programs should also aim to continue ongoing ultrasound quality assurance and improvement during the pandemic to optimize this aspect of patient care.

## CASE RESOLUTION

The emergency physician dons appropriate PPE prior to seeing the patient. She takes a COVID-19 dedicated handheld ultrasound unit and places it in a probe cover. During her assessment, she scans the patient's lungs and identifies focal B lines and subpleural consolidations bilaterally. Because of the patient's initial hypotension, she performs a focused cardiac scan and identifies moderately depressed left ventricular systolic function. In addition to treating the patient's hypotension with intravenous crystalloid, she starts a norepinephrine infusion and urgently gets the patient admitted to the intensive care unit. After the patient assessment, she wipes her gloved hands with disinfectant to decrease the viral load, removes the probe cover with gloves on, and wipes down the probe. She then leaves the patient room, doffs her PPE, and cleans the probe again with a disinfectant wipe.

**Figure:**